# Body Composition Indices and Cardiovascular Risk in Type 2 Diabetes. CV Biomarkers are not Related to Body Composition

**DOI:** 10.1515/med-2020-0043

**Published:** 2020-04-17

**Authors:** Aleksandra Markova, Mihail Boyanov, Deniz Bakalov, Adelina Tsakova

**Affiliations:** 1 Clinic of Endocrinology and Metabolism, University Hospital Alexandrovska, 1 Sv. Georgi Sofiyski str., Sofia 1431, Sofia Bulgaria; 2 Department Internal Medicine, Medical University Sofia, Sofia, Bulgaria; 3 Central Clinical Laboratory, University Hospital Alexandrovska, Sofia, Bulgaria; 4 Department Clinical Laboratory and Clinical Immunology, Medical University Sofia, Sofia, Bulgaria

**Keywords:** Anthropometric indices, NT-proBNP, Asymmetric dimethylarginine, Endothelin-1, Body composition analysis

## Abstract

**Background:**

This study aims to explore the correlations of body mass index (BMI), waist circumference (WC), waist-to-height ratio (WHtR), waist-to-hip ratio (WHR) and body composition with levels of asymmetric dimethylarginine (ADMA), endothelin 1(ET-1), N-terminal brain natriuretic pro-peptide (NT-proBNP) and calculated cardiovascular risks.

**Methods:**

102 women and 67 men with type 2 diabetes participated. Serum levels of NT-proBNP were measured by electro-hemi-luminescence while ELISA were used for ADMA and ET-1. Cardiovascular risks were calculated using the Framingham Risk Score (FRS), the UKPDS 2.0 and the ADVANCE risk engines. Statistical analysis was performed on an IBM SPSS 19.0.

**Results:**

The BMI outperformed all other indices of obesity (WC, WHtR, WHR), as well as body composition parameters (body fat%, fat mass, fat free mass and total body water) in relation to the estimated risks for coronary heart disease and stroke, based on different calculators. The correlations of the obesity indices with the serum cardiovascular biomarkers were not significant except for BMI and fat mass versus ET-1, and for fat free mass and total body water versus ADMA.

**Conclusions:**

The WC, WHR, WHtR, BF%, FM and FFM apparently do not add significant information related to the levels of cardiovascular biomarkers or the calculated CV-risks.

## 1 Introduction

Obesity is one of the leading causes of hypertension, dyslipidemia, diabetes type 2 (T2D) and the resulting atherosclerotic vascular disease. Overweight and obesity can be quantified by simple anthropometric measurements such as the body mass index (BMI), the waist circumference (WC), the waist-to-hip ratio (WHR), the waist-to-height ratio (WHtR) as well as by body composition analysis [1]. Bioelectric impedance analysis (BIA) is one of the simplest and least expensive methods for body composition studies [2]. Its concordance to direct measurement methods such as dual-energy X-ray absorptiometry is very high [3].

Cardiovascular (CV) risk, on the other hand, can be quantified with the use of different calculators. Several CV-risk engines are known worldwide [4], but among the best known are the population-based Framingham Risk Score (FRS) and the diabetes-specific UKPDS or ADVANCE-based calculators [5, 6, 7]. An alternative approach for early detection of atherosclerotic changes might be the measurement of serum biomarkers for endothelial dysfunction and vascular damage [8]. Asymmetric dimethylarginine (ADMA) was shown to be predictive of vascular events in diabetes (coronary artery disease and stroke) [9,10]. Soluble endothelin-1 (ET-1) is increased in diabetes, hypertension, renal failure, and possibly linked to advanced atherosclerotic changes [11]. N-terminal brain natriuretic pro-peptide (NT-proBNP) is a validated marker for heart failure and CV events [12, 13, 14]. These markers are shown to be associated with development and progression of hypertension and the resulting end-organ damage [15]. Hypertension is one of the most prevalent CV risk factors [16,17]. It affects the endothelium through hemodynamic changes, leading to its dysfunction and increased levels of inflammatory markers [15]. T2D and obesity are also linked to higher inflammatory state [18], and in combination with hypertension they might potentiate each other, leading to a greater CV-risk [16].

Few studies have yet explored the relationship of different anthropometric and body composition indices with calculated CV-risks and biochemical markers of CV-risk [19, 20, 21, 22].

The aim of this study was to assess the correlations between anthropometric and body composition indices and the levels of cardiovascular biomarkers (ADMA, ET-1, NT-proBNP), and the calculated CV risks (FRS, UKPDS and ADVANCE based models) in type 2 diabetes patients on oral antidiabetic drugs.

## 2 Methods

### 2.1 Study design and population

This was a cross-sectional observational study. It was approved by the Ethical Committee and the Council for Medical Science at the Medical University, and was conducted according to the international ethical standards and the Declaration of Helsinki. All participants signed informed consent prior to any procedure. They were recruited among the inpatients referred for regular check-up to a tertiary hospital-based University Clinic for Endocrinology and Metabolism. The inclusion criteria were: 1/ age ≥ 18 years; 2/ diagnosed T2D with a duration ≥ 1 year; 3/ diabetes treatment including only oral antidiabetic drugs (OADs) together with diet and lifestyle changes; 4/ willingness to participate. The exclusion criteria included: 1/ type 1 diabetes and symptomatic diabetes secondary to other conditions (endocrine diseases, steroids etc.); 2/ treatment with injectable antidiabetic drugs (GLP-1 preparations, insulin); 3/ evidence or history of previous major CV events (stroke, myocardial infarction, limb amputations) or decompensated heart failure (NYHA stages 3 and 4); 4/ end-stage renal failure or severe hepatic insufficiency.

### 2.2 Measurements and CV risk calculations

Medical history was collected based on the current status and the available previous documentation. Physical examination was performed. Height in cm and weight in kg were measured on a calibrated scale (Tanita Corp., Japan). The BMI was calculated as kg/m^2^. The waist was measured in cm at the umbilical level and the hip circumference - at the widest part of the buttocks or hip. WHR and WHtR were calculated. The blood pressure was measured after 5 minute rest in the sitting position with an aneuroid sphygmomanometer and recorded in mm Hg. A standard 12-lead ECG was performed and read-outs were reviewed and included in the analysis.

Body composition was studied by a leg-to-leg bio-electrical impedance analyzer (Tanita TBF-215 device, Tanita Corp., Japan) using formulas provided by the manufacturer. The results were expressed as follows: fat mass (FM) in kg, body fat percentage (BF %), free fat mass (FFM) in kg, and total body water (TBW) in liters. Precision errors of less than 1 % were reported in a previous study [3].

Morning blood samples were collected after a 12 hour overnight fasting, centrifuged at 4000 rpm for 15 minutes and the supernatant was refrigerated and transferred to the laboratory. Serum levels of NT-proBNP were measured by electro-hemi-luminescence (Elecsys 2010, Roche Diagnostics, Basel, Switzerland), while enzymatic immunoassays were used for measurements of ADMA (BioVendor, Brno, Czech Republic) and ET-1 (IBL International GMBH, Hamburg, Germany). The glycemic and metabolic parameters were assessed by routine methods: glycated hemoglobin A1c in % and mmol/mol, lipid profiles (total cholesterol, HDL-cholesterol, calculated LDL-cholesterol, triglycerides in mmol/l), serum creatinine in μmol/l, urine measurements for albumin/creatinine ratio (ACR in mg/mmol/l and μg/mg) in a fresh morning urine sample. Estimated glomerular filtration rate (eGFR) was calculated in ml/min/1.72 m^2^ using the Modification of Diet in Renal Disease (MDRD) formula. Mean daily blood glucose was calculated from 4-point measurements (fasting morning, pre-lunch, 2hr post-lunch and bedtime BG).

Cardiovascular risks were calculated using the Framingham Risk Score (FRS), the UKPDS version 2.0 and the ADVANCE risk engines. The FRS was selected as one of the most popular general population-based tools, while the UKPDS and the ADVANCE calculators – as very well validated diabetes-specific tools. All three engines calculated absolute risks in % for a different period of time. The FRS issued a 10-year risk in % combining all major CV-outcomes. The UKPDS risk engine calculated separate 10-year risks for coronary heart disease (CHD), fatal CHD, stroke and fatal stroke. The ADVANCE based calculations produced a composite 4-year CV-risk.

### 2.3 Statistical analysis

All analyses were performed on an IBM SPSS 19.0 for Windows platform (SPSS Corp., Chicago, IL). The sample size was selected based on a theoretical minimum of 150 participants allowing correlation and regression analyses. Descriptive statistics and frequency analysis were performed. Data were checked for normal distribution based on their skewness and curtosis, as well as on Q-Q plots. Most of the data were positively skewed including the biochemical markers and the calculated CV-risks. Medians, percentiles and quartile ranges were used. Correlation analysis was performed and the corresponding Spearman’s Rho recorded. The regression analyses included 10 curves (linear, quadratic, cubic, power, S, inverse, exponential, log models, growth, compound models) and the best models were selected based on the highest R^2^ and the lowest p-value. Statistical significance was set as p<0.05. A power analysis was performed post hoc and revealed significance of the study based on already known correlation/regression coefficients and a confidence interval of 95%. The minimal number of participants was 150 for statistical power of this study.

## 3 Results

### 3.1 Clinical, anthropometric and laboratory data of the participants

102 women and 67 men with type 2 diabetes met the inclusion/exclusion criteria and agreed to participate in this cross-sectional study. Their mean age was 60.3 ± 9.6 years (range - 33 – 83); the mean diabetes duration – 7.6 years. The mean body weight was 88.9 ± 20.3 kg (41 – 156 kg), the BMI - 32.8 ± 6.1 kg/m^2^ (19.3 – 53.2), the WC - 107.5 ± 14.0 cm (74 – 143), the WHR -0.97 ± 7.93 (0.81 – 1.21) and the WHtR – 0.64 ± 0.09 (0.45 – 0.88). The mean BF% was 40.3 ± 7.9 %, the FM – 36.4 ± 12.5 kg, the FFM – 52.2 ± 11.0 kg, and the TBW – 37.4 ± 7.4 kg. The mean systolic blood pressure (SBP) was 136.0 ± 16.7 mm Hg (100 - 210), while the diastolic blood pressure (DBP) - 83.6 ± 9.6 mm Hg (60 - 120).

The mean glycated hemoglobin A1c was 7.9 ± 1.9 % (DCCT), or 62 ± 9 mmol/mol (IFCC). The mean LDL-cholesterol was 3.03 ± 1.06 mmol/l, the triglycerides were 2.04 ± 1.47 mmol/l. The estimated glomerular filtration rate (eGFR) as calculated by the MDRD formula was 87.1 ± 23.5 ml/min/1.73 m^2^. The albumin-to-creatinine ratio (ACR) was 9.6 ± 40.6 in mg/mmol/l, and 84.7 ± 388.3 in μg/mg.

The median values and interquartile ranges of the three biochemical markers (ADMA, ET-1 and NT-proBNP) and the calculated CV-risks (FRS, UKPDS, ADVANCE) are summarized in Table 1.

**Table 1:** The biochemical markers of cardio-vascular risk / endothelial dysfunction and the calculated cardio-vascular risks are presented as medians and interquartile ranges (skewness and curtosis were both positive).

	25th percentile	50th percentile	75th percentile
**Biomarkers for CV-risk**			
ADMA (μmol/l)	0.54	0.60	0.67
Endothelin-1 (pg/ml)	5.45	11.70	23.35
NT-proBNP (pmol/l)	3.92	8.75	20.14
**CV risk calculations**			
4-yr ADVANCE based risk (%)	1.38	2.65	4.95
UKPDS-based 10-yr risk for CHD (%)	11.03	16.55	25.20
UKPDS-based 10-yr risk for fatal CHD (%)	6.53	10.95	17.00
UKPDS-based 10-yr risk for stroke (%)	4.33	8.15	13.38
UKPDS-based 10-yr risk for fatal stroke (%)	0.60	1.20	2.00
Framingham risk score - 10-yr risk (%)	3.10	6.20	12.60

### 3.2 Correlation analyses linking the biochemical/endothelial markers and the calculated cardiovascular risks with the anthropometric and body composition indices

The results of the analyses correlating the biochemical/endothelial markers and the calculated cardiovascular risks with the anthropometric and body composition indices are presented in Table 2.

**Table 2:** The correlation and coefficients and regression models linking the anthropometric and body composition indices (independent variables) with the biochemical/endothelial markers and the calculated cardiovascular risks (dependent variables) are shown. The significant Spearman’s Rho Rank coefficients are shown.

Independent variables	ADMA (μmol/l)	ET-1 (pg/ ml)	NT- proBNP (pmol/l)	ADVANCE- based 4-yr risk (%)	UKPDS- based 10-yr risk for CHD (%)	UKPDS-based 10-yr risk for fatal CHD (%)	UKPDS-based 10-yr risk for stroke (%)	UKPDS- based 10-yr risk for fatal stroke (%)	Framingham Risk Score – 10-yr risk (%)
Correlation coefficients									
BMI (kg/m^2^)	N/A^a^	Rho^b^=-0.163	N/A	Rho=0.231	Rho=-0.237	Rho=-0.264	Rho=-0.259	Rho=-0.190	N/A
		p=0.048		p=0.048	p=0.003	p=0.001	p=0.001	p=0.019	
WC (cm)	N/A	N/A	N/A	N/A	N/A	N/A	N/A	N/A	N/A
WHtR	Rho=0.269	N/A	N/A	Rho=-0.249	Rho=-0.223	Rho=-0.216	Rho=-0.208	N/A	N/A
	p=0.006			p=0.050	p=0.031	p=0.036	p=0.045		
WHR	N/A	N/A	N/A	N/A	N/A	N/A	N/A	N/A	Rho=0.324
									p=0.034
BF%	N/A	N/A	N/A	N/A	Rho=-0.243	Rho=-0.211	Rho=-0.214	N/A	Rho=-0.312
					P=0.015	p=0.035	p=0.033		p=0.001
FM (kg)	N/A	Rho=-0.215	N/A	N/A	N/A	Rho=-0.194	Rho=-0.305	Rho=-0.260	N/A
		p=0.040				p=0.050	p=0.002	p=0.009	
FFM (kg)	Rho=-0.221	N/A	N/A	N/A	N/A	N/A	Rho=-0.277	Rho=-0.227	Rho=0.238
	p=0.029						p=0.007	p=0.028	p=0.020
TBW (kg)	Rho=-0.314	N/A	N/A	N/A	N/A	N/A	Rho=-0.271	Rho=-0.222	N/A
	p=0.003						p=0.013	p=0.043	
Regression models									
BMI (kg/m^2^)	N/A	Inverse, R^2^=0.026, F=3.84, p=0.05	N/A	Compound, R^2^=0.064, F=4.89, p=0.030 + 4 others^c^	Cubic, R^2^=0.086, F=4.65, p=0.004 + 9 others	Cubic, R^2^=0.094, F=5.09, p=0.002 + 9 others	Compound, R^2^=0.047, F=7.46, p=0.007 + 4 others	Compound, R^2^=0.030, F=4.56, p=0.034 + 4 others	N/A
WC (cm)	N/A	N/A	N/A	N/A	N/A	N/A	N/A	N/A	N/A
WHtR	Inverse, R^2^=0.053, F=5.66, p=0.019 + 7 others	N/A	N/A	Quadratic, R^2^=0.108, F=3.59, p=0.034 + 4 others	N/A	N/A	N/A	N/A	N/A
WHR	N/A	N/A	N/A	N/A	N/A	N/A	N/A	N/A	Cubic, R^2^=0.155, F=3.67, p=0.034 + 4 others
BF%	N/A	N/A	N/A	N/A	N/A	Cubic, R^2^=0.134, F=4.93, p=0.003 + 9 others	Cubic R^2^=0.092, F=3.23, p=0.026 + 8 others	N/A	N/A
FM (kg)	N/A	N/A	N/A	N/A	N/A	Inverse, R^2^=0.070, F=7.36, p=0.008 + 4 others	Power, R^2^=0.089, F=9.56, p=0.003 + 7 others	Compound, R^2^=0.063, F=6.55, p=0.012 + 6 others	N/A
FFM (kg)	Quadratic, R^2^=0.068, F=3.44, p=0.036 + 8 others	N/A	N/A	N/A	N/A	N/A	S-model, R^2^=0.049, F=4.51, p=0.032 + 4 others	N/A	Cubic, R^2^=0.094, F=3.15, p=0.029 + 9 others
TBW (kg)	Power, R^2^=0.099, F=9.26, p=0.003 + 9 others	N/A	N/A	N/A	N/A	N/A	N/A	N/A	N/A

a N/A – no significant correlations or regression models available ^b^ Rho - Spearman’s rank correlation Rho (non-parametric) ^c^ +others – multiple models available; the strongest one is described

Table 2 shows that BMI is the obesity index that is related to most of the calculated CV risks and to serum ET-1 additionally. The WC and WHR are practically not related to any of the variables under study. The WHtR is related only to risks based on the ADVANCE and UKPDS calculators, while BF% - to risks based on UKPDS and FRS. FFM and TBW correlated only to the UKPDS-based risks for stroke, with FFM being correlated with the FRS additionally. This tendency was seen once more in the regression analyses. The most often fitting model was the cubic one, followed by the compound model. Again, different regression equations were significant showing a complex pattern of the interrelationships.

Multiple regression analysis was not performed as the data did not meet the required statistical assumptions. The relationship between independent variables (anthropometric measurements and body composition indices) and the dependent variables (calculated CV-risks, serum levels of biomarkers) was not linear (see Table 2). The variances were unequal and the error distribution was not normal. The attempt to transform the data, to correct their and to achieve curvilinear effects was unsuccessful.

## 4 Discussion

The main purpose of our study was to determine the association of a few obesity indices, reflecting the body composition and fat distribution, with serum biomarkers of endothelial dysfunction and calculated CV - risk scores in a cohort of T2D patients on oral antidiabetic drugs. Our main hypothesis was that increasing obesity and visceral adiposity would correlate positively with the levels of ADMA, ET-1 and NT-proBNP and the CV-risk calculations, using FRS, UKPDS and ADVANCE engines. Our data showed, however, that the BMI followed by the WHtR and the BF% showed the strongest associations with the calculated cardiovascular risk estimates, while FM, FFM and TBW performed quite worse and were associated only with the risks for stroke. Levels of NT-proBNP were not related to any measure of obesity, while ADMA showed links to WHtR, FFM and TBW, and ET-1 – to BMI and FM only. We found an inverse correlation of obesity with CV-risks and biomarkes. Our analyses showed that the relationship of obesity and body composition indices with the CV biomarkers and calculated risks is not linear and the association might be an inverse one, a finding that seems counter-intuitive. It implies that other factors arising from obesity or comorbidities might have stronger influence on the atherosclerotic process and the subsequent vascular events. There are inflammatory mediators associated with obesity and T2D, such as inteleukin-6 (IL-6), IL-32, tumor necrosis factor – α (TNF-α) [18], that have a direct effect on vascular wall and plaque formation [23], but they are not within the scope of this work. To note that the correlations of the obesity measures with the levels of the CV biomarkers (ADMA, ET-1 and NT-proBNP) are practically not significant. This finding could be interpreted in a sense that obesity needs some time to change the CV risk profile which is not mirrored by the biomarkers as early witnesses of endothelial damage and heart load.

### 4.1 Association of obesity indices, body composition and biochemical markers

Scarce data from the literature indicate that ADMA is increased in obesity or the metabolic syndrome [24]. In healthy young adults ADMA showed a positive association with body weight, BMI, WC, WHtR and body FM studied by BIA [25]. A positive correlation was found between ADMA and body mass in healthy 1-year old children [19]. ADMA was a correlate of insulin resistance, BMI and WC in early-stage diabetes, while the only correlation in non-diabetic controls was with the WC [20,26]. Therefore, it is a little surprising that in our patients ADMA was inversely correlated with the FFM and TBW. This is a finding needing further investigations.

In our study ET-1 was linked to BMI and FM only. Overweight and obese subjects were found to have increased ET-1 levels [27] while weight reduction could lead to a decrease in its levels (28). ET-1 has been linked to BMI as an index of obesity [29], although another study did not corroborate this finding in adolescents [21,22].

To our surprise, the levels of NT-proBNP did not correlate to any of the obesity indices of our patients. As a marker of heart failure it has been extensively studied and showed an inverse relationship with body weight [21,30,31]. The inverse relationship of NT-proBNP with BMI, WC and BF% was confirmed in a few studies [32], although others put more weight on lean mass, not on BF% [21]. Some relationships were best described by a U-shape, such as that of NT-proBNP with WC [33].

### 4.2 Association of obesity indices, body composition and CV-risk calculations (UKPDS, FRS and ADVANCE risk engines)

The UKPDS-based CV risk calculations were consistently related only to the BMI and WHtR, while BF% was related mainly to the risk for CHD (fatal or not), and the FM, FFM and TBW – mainly to the risk for stroke (fatal or not). A study tested the adiposity markers versus the UKPDS-based risk of CHD in type 2 diabetes patients and found that the associations of weight, BMI and WHR did not hold in their sample [34, 35, 36, 37]. More publications included FRS-based risk calculations [34]. In a sample of Chinese women the WHtR was best correlated to the FRS-based risk [35]. WHtR was found as the best predictor of FRS-based calculations in another study, followed by the WC [36]. Another study of FRS in high-risk ethnic groups highlighted the best predictive value of WHR, while the WC was linked best to the arterial stiffness, and BMI was of value in specific subgroups only [37]. In a DXA-based study, the FRS estimated higher 10-yr CVD risk was associated with a combination of low muscle mass and high fat in men but not in women [38]. In the BIA-based PREVEND study BMI and BF% remained independently associated with CV events in both men and women, while WC – in men only, but not in women [38]. In our sample the 10-yr risk according to the FRS was associated only with the WHR, BF% and FFM, which is an ambiguous finding. We found no data in the literature on the relationship of obesity indices with the ADVANCE-based risk calculations, but in our sample only the BMI and WHtR correlated with these estimations.

### 4.3 Strengths and limitations

The major strength of our study is that we correlated the obesity indices with two different approaches for CV risk estimation – the first one revealing early vascular damage (biomarkers), while the second one being based on specific risk calculators and forecasted events.

This study however, has a number of inherent limitations. First, it was a cross-sectional one and quite moderate in size. Secondly, the included diabetes patients were on OADs only, which might have introduced a bias for shorter duration and less severity of vascular complications of the diabetes itself. Thirdly, we used risk calculations based on epidemiological models coming from other populations and not validated in Bulgarian participants.

### 4.4 Conclusions

Among the different indices of obesity, the BMI outperformed all others (the WC, WHtR, WHR, as well as body composition parameters such as BF%, FM, FFM and TBW) in relation to the estimated risks for CHD and stroke based on specific calculators. The correlations of those obesity indices with the serum biomarkers were not significant except for BMI and FM versus ET-1, and FFM and TBW versus ADMA. Measuring the WC, WHR, WHtR or body composition does apparently not add significant information correlated to the serum/plasma levels of markers of endothelial dysfunction and vascular damage or to the calculated CV-risks. More robust studies are needed to define the optimal contribution and possibly the combined use of obesity indices in the CV-risk estimation.

## Abbreviations

ADMA: assymetric dymethylarginine
ADVANCE: PreterAx and DiamicroN Controlled Evaluation Study
BF%: body fat percent
CHD: coronary heart disease
CVD: cardiovascular disease
ET-1: endothelin 1
FFM: fat-free mass
FM: fat mass
FRS: Framingham Risk Score
NT-proBNP: N-terminal brain natriuretic pro-peptide
OAD: oral antidiabetic drugs
T2D: type 2 diabetes
TBW: total body water
UKPDS: United Kingdom Prospective Diabetes Study
WC: waist circumference
WHtR: waist-to-height ratio
WHR: waist-to-hip ratio
